# Inferring a Property of a Large System from a Small Number of Samples

**DOI:** 10.3390/e24010125

**Published:** 2022-01-14

**Authors:** Damián G. Hernández, Inés Samengo

**Affiliations:** Department of Medical Physics, Centro Atómico Bariloche and Instituto Balseiro, San Carlos de Bariloche 8400, Argentina

**Keywords:** inference, bayesian, undersampled, entropy, mutual information

## Abstract

Inferring the value of a property of a large stochastic system is a difficult task when the number of samples is insufficient to reliably estimate the probability distribution. The Bayesian estimator of the property of interest requires the knowledge of the prior distribution, and in many situations, it is not clear which prior should be used. Several estimators have been developed so far in which the proposed prior us individually tailored for each property of interest; such is the case, for example, for the entropy, the amount of mutual information, or the correlation between pairs of variables. In this paper, we propose a general framework to select priors that is valid for arbitrary properties. We first demonstrate that only certain aspects of the prior distribution actually affect the inference process. We then expand the sought prior as a linear combination of a one-dimensional family of indexed priors, each of which is obtained through a maximum entropy approach with constrained mean values of the property under study. In many cases of interest, only one or very few components of the expansion turn out to contribute to the Bayesian estimator, so it is often valid to only keep a single component. The relevant component is selected by the data, so no handcrafted priors are required. We test the performance of this approximation with a few paradigmatic examples and show that it performs well in comparison to the ad-hoc methods previously proposed in the literature. Our method highlights the connection between Bayesian inference and equilibrium statistical mechanics, since the most relevant component of the expansion can be argued to be that with the right temperature.

## 1. Introduction

Systems with a large number of states appear ubiquitously in the era of big data. Often, the number of available observations—which, in this paper are referred to as “samples”—is smaller than the number of states, sometimes considerably smaller. Although in these circumstances estimating the full probability distribution of the system is highly unreliable, here we show that even in the severe undersampled regime, fairly accurate inferences are often still possible. The clue is that, if only a single property of the system needs to be inferred, or maybe just a few properties, the complete description of the probability distribution is unnecessary. Examples of desired properties may be, for instance, the information processing capacity, the correlation between pairs of variables, or the diversity of reachable states in the system.

Let *k* be the number of available states of a discrete system, and $q=(q_{1},\cdots ,q_{k})$ the (unknown) probability distribution that governs the occupation of the states. The number of samples *n* is assumed to be small compared with the effective number of states exp[H(q)], where H(q) is the entropy of the system. Still, *n* is assumed to be large enough so that, occasionally, some of the states are sampled more than once. In mathematical terms, this means that *n* is at least as large as the square root of the effective number of states; that is, $n≳\sqrt{\exp[H(q)]}$ [1]. For even smaller samples, most of the states remain unsampled, and the few states that are sampled are likely to get no more than a single sample. When *n* reaches the order of $\sqrt{\exp[H(q)]}$, the probability that a small fraction of the states get more than a single sample becomes large [1]. We are interested in estimating a property $F\left(q\right)=F\left(q_{1},\cdots ,q_{k}\right)$ whose functional form in terms of q is known. The naïve approach—which we here discourage—would be to first estimate q and then evaluate F(q) in the estimated value. This strategy is called the “plug-in” estimator. Although it may work for properties that depend linearly on the probabilities, it is often appallingly biased in nonlinear cases—in particular, for information-related properties that typically bear a logarithmic dependence on $q_{i}$. To overcome this problem, some idiosyncratic solutions have emerged during the last decades that are valid for particular properties, such as entropy [2,3,4,5,6] and mutual information [7,8,9].

Here, we develop a principled way to approach this problem for properties F(q) that may be arbitrary, as long as they vary smoothly with q. The clue relies on the fact that the simplex Q containing the set of possible q-vectors can be foliated by the level surfaces of F(q). If only the value of *F* matters, estimating the full q is excessive; the only inference that needs to be made is about the level surface to which q belongs. This is a one-dimensional inference problem and hence is much simpler than the *k*-dimensional problem of estimating q. We here estimate *F* within a Bayesian framework, using a prior distribution that weighs the different level surfaces according to a MaxEntropy principle.

The main contributions of the paper are as follows:

- A new method to construct Bayesian estimators is proposed;
- The method is applicable to arbitrary quantities F(q);
- A conceptual framework is provided, in which the one-dimensional nature of the problem is exploited;
- The search of the prior is restricted to the level surfaces of F(q);
- The Maxentropy principle is employed to capitalize both what we know and what we do not know about the prior;
- The performance of the method is tested in known examples;
- The conceptual connection with equilibrium statistical mechanics is highlighted.

The paper is organized as follows. In the next section, the theoretical Bayesian framework is described in general: firstly, pointing out that only certain aspects of the prior matter in the inference of the property; secondly, using the symmetries of the property and our ignorance about the underlying distribution to constrain the search for the prior; thirdly, by finally constructing the prior with a MaxEntropy principle; and finally, showing how this approach relates to equilibrium statistical mechanics. In the third section, the method is applied to different example properties, including mutual information and entropy. Finally, in the last section, the implications and perspectives of the work are discussed and summarized.

## 2. Rationale of the Inference Process

Section 2 develops the theoretical underpinnings of the new estimator proposed in this paper.

### 2.1. A Bayesian Approach to the Inference Problem

In this subsection, we define the estimator to be used and formulate the problem within the Bayesian framework. The search for the prior is established as the main target of the paper.

**Figure 1:**
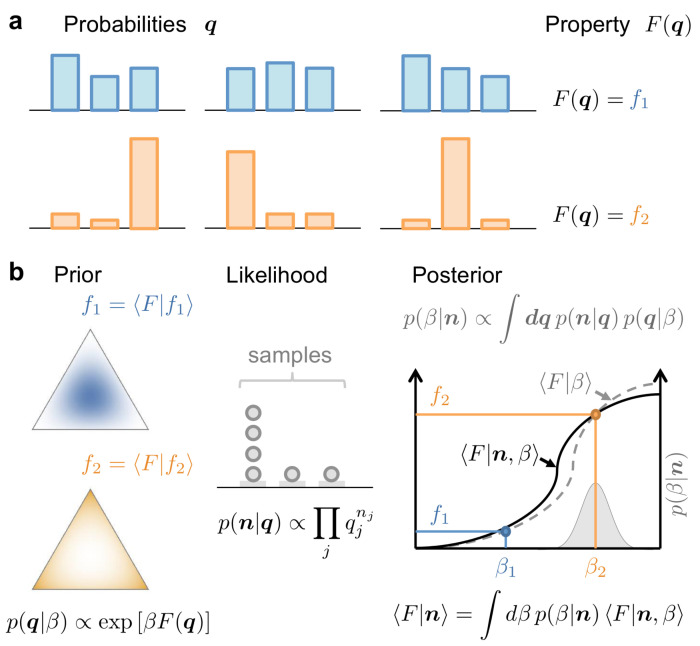
Conceptual framework used to estimate the property F(q) of a stochastic system with probabilities q from a limited number of observations, or samples n. (**a**) Several probabilities q can produce the same value of the property. All such q-vectors belong to the same level surface of F(q). (**b**) Left: Example case in which the property F(q) has circular level surfaces on the simplex embedded in three-dimensional space. Two members of the family of base functions used to expand the prior are shown, containing the different q vectors shown in (**a**). These two members are solutions of the Maxentropy problem with two different expected values *f* of the property. Middle: The histogram n generated by the sampled data produces a likelihood p(n|q) that selective favors a specific range of surface levels. Right: The most favored *f* value (or equivalently, β value) is the one for which the prior and posterior estimates of the property coincide.

Our goal is to estimate a property F(q) of a stochastic system composed of a large number *k* of states, when we only have access to *n* samples, with $n≳\sqrt{\exp[H(q)]}$. The probabilities $q=(q_{1},\cdots ,q_{k})$ constitute a complete description of the system (see Figure 1).

We choose to work with the estimator $\hat{F}=\langle F\left(q|n\right)\rangle$ defined as the expected value of the property F(q) when conditioned by the measured data n. The average 〈·〉 is calculated with the posterior distribution p(q|n), and the *k*-dimensional vector n has components $n_{1},\cdots ,n_{k}$, with $n_{i}$ equal to the number of samples in which the system was observed in the *i*th state. Therefore,

(1) $$\hat{F}=\langle F|n\rangle =\int dq\;F\left(q\right)\;p\left(q|n\right).$$

Throughout the paper, all integrals in q should be restricted to the space where q is defined: the simplex embedded in $R^{k}$, or the cartesian product of several simplexes, in the multivariate case. The estimator of Equation (1) minimizes the mean square error of the estimation [10]. Using Bayes’ rule, the posterior can be written in terms of the likelihood p(n|q) and the prior p(q), so that

(2) $$\langle F|n\rangle =\int dq\;F\left(q\right)\;\frac{p(n|q)p(q)}{p(n)}.$$

The likelihood p(n|q) is the only factor that depends on the sampled data n, and for discrete states, it is a multinomial distribution, namely

$$p\left(n|q\right)\propto \prod_{j}q_{j}^{n_{j}}.$$

The only factor that still needs to be defined is the prior p(q), and the strategy underlying this choice is the topic of this paper. We want the prior to produce an inductive bias that acknowledges our ignorance about F(q). Such bias will be useful to overcome the scarcity of samples, but it will only be suitable to estimate the specific F(q) of the problem at hand and not others. In other words, the prior proposed here is individually tailored for F(q).

### 2.2. Defining the Prior on the Level Surfaces of the Property

In this subsection, the apparent high-dimensionality of the problem is shown to be illusory. The prior we are searching for, although defined in a high-dimensional simplex, is here shown to affect the result of the inference process only through its integrated value inside each level surface of the quantity F(q). In other words, the variations of p(q) inside the level surfaces of F(q) are irrelevant since they have no influence on the value of the estimator $\hat{F}$. These level surfaces can be indexed by a single-dimensional parameter, which will later allow us to drastically simplify the search for the prior.

To show this point, we start by noting that p(q) is defined on the simplex embedded in $R^{k}$. We introduce a change of variables $q\to q^{\prime }$ to perform the integral of Equation (2). Specifically, the first two coordinates of the new variables are chosen to be

(3) $$\begin{array}{ccc} q_{1}^{\prime }\left(q\right) & = & f=F(q)=\mathrm{property}\;\mathrm{to}\;\mathrm{be}\;\mathrm{inferred}\\ q_{2}^{\prime }\left(q\right) & = & \ell =q(n|q)=\mathrm{likelihood}\;, \end{array}$$

and the remaining coordinates $q_{3}^{\prime }\left(q\right),\cdots ,q_{k}^{\prime }\left(q\right)$ may be chosen arbitrarily, as long as the transformation $q\to q^{\prime }$ be invertible. In the new variables,

(4) $$\begin{array}{ccc} \langle F|n\rangle & = & \int df\;f\;\int d\ell \;\ell \int dq_{3}^{\prime }\cdots dq_{k}^{\prime }\;\frac{p(q^{\prime })}{p(n)}\;\left|\frac{\partial q_{1},\cdots ,q_{k}}{q_{1}^{\prime },\cdots ,q_{k}^{\prime }}\right|\\ & = & \int df\;f\;\int d\ell \;\ell \;p(f,\ell |n),\;\;\;\;\mathrm{with}\\ p(f,\ell |n) & = & \int dq_{3}^{\prime }\cdots dq_{k}^{\prime }\;\frac{p(q^{\prime })}{p(n)}\;\left|\frac{\partial q_{1},\cdots ,q_{k}}{q_{1}^{\prime },\cdots ,q_{k}^{\prime }}\right|\\ & = & \int dq\;\frac{p(q)}{p(n)}\;\delta \left[f\left(q\right)-f\right]\;\delta \left[L\left(q\right)-\ell \right]. \end{array}$$

Equation (4) shows that it does not matter how the prior p(q) distributes the density inside the manifold $M_{f\ell }$ obtained by intersecting the level surface *f* of the property *F* with a level surface *ℓ* of the likelihood *L*. Only the integral of q inside $M_{f\ell }$ has an effect on $\hat{F}$. Therefore, we opt to consider only priors p(q) that are constant inside $M_{f\ell }$, since for any prior not constant in $M_{f\ell }$, a prior that is constant in $M_{f\ell }$ exists that produces the same estimation $\hat{F}$. Our task is therefore to design how the prior p(q) changes with *f* and *ℓ*. A crucial point is that the level surfaces of L(q) vary with the data n. Yet, by definition, a prior cannot depend on the measured data. Therefore, we only work with priors that, when written in the q-coordinates, only depend on q through F(q). In other words, we assume that a function *g* exists such that p(q)=g[F(q)]. By limiting the search to priors of this type, for each value of *f*, we impose maximal uncertainty about q, as dictated by a maxentropy principle.

As a side remark, we point out that the assumption p(q)=g[F(q)] does not imply that, when written in the $q^{\prime }$-coordinates, the prior $p(q^{\prime })$ must be independent of $\ell =q_{2}^{\prime }$, since the Jacobian of the transformation of variables may well depend on *ℓ*. Moreover, the marginal prior p(f,ℓ|n) may also depend on *ℓ*, since the level surface in which Equation (4) is calculated depends on L(q). Therefore, p(f,ℓ|n) is allowed to depend on *ℓ*, but only due to the geometrical structure that the delta function in L(q) restricts the integration region of Equation (4).

### 2.3. Decomposing the Prior as a Linear Combination of a Family of Base Priors

In this subsection, we exploit the fact that p(q)=g[F(q)] to decompose the prior as a linear combination of a collection of base priors indexed by a single real number *f*. The decomposition allows us to reduce the search of a prior p(q) defined in a high-dimensional simplex to the search of a density g(f) defined as a function of *f*. This step brings about a dramatic simplification, but more importantly, it reduces the relevance of the subjective aspect involved in the selection of priors. We show here that the range of *f*-values that are relevant to the inference process (and thereby, the base priors that actually matter) is only partially defined by g(f), which is the only element still containing subjective choices. In many applications, the sampled data *n* will turn out to be the truly defining factor in selecting the base priors that participate in the inference process.

We start to demonstrate these statements by noting that the set of priors p(q) for which a function g:R→R exists such that p(q)=g[F(q)] is equal to all the distributions of the form

(5) p(q)=∫dfg(f)δ[F(q)−f]

that can be obtained by varying g(f). Equation (5) implies that the problem of specifying p(q) can be reduced to the problem of specifying g(f), which is a drastically simpler object, given that q has (k−1) dimensions, whereas *f* has only a single dimension.

We now show that, when the number of samples is sufficiently large, the determination of *g* becomes unnecessary, since for large *k*, the measured data n often enter into the likelihood in such a way that only a small range of *f*-values remains with significantly non-zero probability. Therefore, all the *g*-functions that do not vary drastically within the permitted range give rise to the same estimation. In such cases, the data alone dictate the value of the estimator through the likelihood, making the discussion about priors essentially inconsequential. We now prove these statements. Replacing Equation (5) in (2), the estimator becomes

(6) $$\begin{array}{ccc} \langle F|n\rangle & = & \frac{1}{p(n)}\;\int dq\;p\left(n|q\right)\;F\left(q\right)\;g\left[F\left(q\right)\right]\;\delta \left[F\left(q\right)-f\right]\\ & = & \int df\;f\;p(f|n) \end{array}$$

with

(7) $$\begin{array}{ccc} p(f|n) & = & \frac{g(f)}{p(n)}\;\int dq\;p\left(n|q\right)\;\delta \left[F\left(q\right)-f\right]\\ & \propto & \frac{g(f)}{p(n)}\;\int dq\;e^{-n\;D_{\mathrm{KL}}(\frac{1}{n}n||q)}\;\delta \left[F\left(q\right)-f\right], \end{array}$$

where $D_{\mathrm{KL}}\left(a||b\right)$ is the Kullback–Leibler divergence [11] between the distributions a and b, and the *i*th component of the vector $\frac{1}{n}n$ is $n_{i}/n$. The divergence is always non-negative, and it only vanishes when the two distributions coincide [12]. Therefore, if the number of samples *n* is sufficiently large, the result of the integral is significantly different from zero only for level surfaces that pass close enough to the sampled frequencies n/n and is maximal for the one that contains the sampled frequencies. The data n thereby select the range of *f* values that are compatible with the observations. The factor *n* in the exponent of Equation (7) implies that the allowed range of *f*-values becomes increasingly narrow as the number of samples grows. The crucial point in this reasoning is that for sufficiently narrow ranges, the shape of the prior g(f) becomes irrelevant. If the range is much narrower than the typical scales in which g(f) varies, for all practical matters, g(f) is constant within this range, and has no bearing in the estimation. This is the situation in which we no longer need to bother about the prior.

When that limit is reached, the likelihood becomes a delta distribution, and the only q-vector that contributes to a given *f*-value is q=n/n. In this extreme case, when g(f) is uniform, the estimator 〈F|n〉 converges to the plug-in estimator. Yet, the plug-in estimator is only justified when the likelihood indeed reaches the delta-like behavior, which only happens for n≫k. Before this limit, the plug-in estimator completely neglects the width of the peak of the likelihood around the sampled frequencies n/n and thereby the possibility that a whole collection of q-vectors in the vicinity of n/n contribute to each *f*. Allowing for this possibility is important since, in the undersampled regime, there is a large degree of uncertainty about the true q that generated the data n. By neglecting this uncertainty, the plug-in estimator produces the undesired biases mentioned before. The obvious solution is not to neglect the width of the likelihood. However, if no approximations are made, it is difficult to calculate or even to estimate p(f|n) analytically, given that Equation (7) involves an integral over an arbitrarily shaped manifold.

To overcome this problem, we would like to design another estimator that retains insensitivity to the prior but still gives a chance for a collection of q vectors to contribute to each *f*. The size of this collection is determined by the width of the likelihood; that is, by the total number of samples *n*, and the obtained frequencies n/n. The averaged contributions of all the q vectors that have non-vanishing likelihoods produces an estimated $\hat{F}$ that may be either smaller or larger than the plug-in value. In other words, all the q-vectors different from n/n may tend to either increase or decrease *F* as compared with F(n/n), and the direction and size of this shift depends on the behavior of F(q) around q=n/n. In what follows, we propose an alternative expansion of the prior different from Equation (5), which is formulated in terms of a parameter that controls the relative weight of the level surfaces that tend to increase vs. those that tend to decrease *F*.

Using the previous analysis as an inspiration, from now on, we consider a narrower set of priors that is a proper subset of the one defined by Equation (5). A restriction in the set of possible priors does not invalidate the analysis of how the width of the likelihood depends on the sampled data, it only reduces the set from which priors are selected. We begin by proposing the decomposition

(8) p(q)=∫dfp(q|f)g(f),

for a conveniently chosen family of priors p(q|f) that now need not coincide with δ[F(q)−f] but are still assumed to only depend on q through the property F(q). In other words, we assume that a family of normalizable functions $g_{f}:R\to R$ exists such that

$$p\left(q|f\right)=g_{f}\left[F\left(q\right)\right].$$

Before continuing, it is important to note that in the new decomposition of Equation (8), the parameter *f* can no longer be identified as exactly the value of the property F(q) in a given level surface, since the delta functions are no longer present to restrict p(q|f) to a single level surface of F(q). Therefore, from now on, *f* should be regarded as a formal label that parametrizes the set of base functions used in the expansion. Yet, later on, we choose a family of base functions for which there is still a connection between *f* and F(q), but the connection turns out to be probabilistic (see next subsection).

By inserting Equation (8) in Equation (2), the estimator becomes

$$\begin{array}{ccc} \langle F|n\rangle & = & \int dq\;F\left(q\right)\;\frac{p(n|q)}{p(n)}\;\int df\;p\left(q|f\right)\;g\left(f\right)\\ & = & \int df\;\frac{g(f)}{p(n)}\;p\left(n|f\right)\;\int dqF\left(q\right)\;p\left(n|q\right)\;\frac{p(q|f)}{p(n|f)} \end{array}$$

where we have introduced the likelihood of the data for each value of the parameter,

(9) p(n|f)=∫dqp(q|f)p(n|q).

With this definition, the estimator reads

(10) 〈F|n〉=∫dfp(f|n)〈F|n,f〉,

where

(11) $$p\left(f|n\right)=\frac{p(n|f)\;g(f)}{p(n)}$$

represents the amount of evidence in favor of each *f*-value provided by the measured data n, and

(12) $$\begin{array}{ccc} \langle F|n,f\rangle & = & \int dq\;F(q)\;\;p(q|n,f)\\ & = & \int dq\;F\left(q\right)\;p\left(n|q\right)\;\frac{p(q|f)}{p(n|f)}. \end{array}$$

is the estimation of the property F(q) conditional on the measured data n and the parameter *f*.

Equation (10) is homologous to Equation (6) for base priors that are not delta distributions. Using Bayes’ rule (Equation (11)), the evidence p(f|n) can be written in terms of the marginal likelihood p(n|f) of Equation (9). This evidence is defined by an integral in q-space that contains the multinomial likelihood p(n|q) embodying the Kullback–Leibler divergence. Therefore, just as before, the data n still select a range of *f*-values; only now, we cannot identify *f* as an instantiation of *F* on one specific region. Still, the value of *f* that maximizes the posterior p(f|n) is the one that makes the largest contribution to the integral in Equation (10). Before, only keeping the optimal *f*-value yielded to almost the plug-in estimator (except for the effect of g(f)). In the present case, only keeping the optimal *f*-value means to replace the integral in Equation (10) by the evaluation of 〈F|n,f〉 at the value of $f=f^{*}$ that maximizes p(f|n). This procedure is henceforth denominated the *MAP estimator*, for *Maximum A Posteriori*. With the present decomposition, this procedure does not yield the plug-in estimator, because $\langle F|n,f^{*}\rangle$ contains an integral that sweeps through a whole range of level surfaces. For the family of base priors selected in the following subsection, the MAP estimator performs substantially better than the plug-in estimator and often also better than custom-made estimators designed for specific properties. In those cases in which the shape of g(f) can be argued to play no relevant role, the MAP estimator can be replaced by an empirical Bayes estimator, in which the selected $f^{*}$ value maximizes the marginal likelihood p(n|f) instead of the marginal posterior p(f|n).

### 2.4. A Maxentropy Strategy to Select the Base of Functions to Expand the Prior

In this subsection, we select the family of distributions p(q|f) that serve as a base to expand the prior p(q), as dictated by Equation (8). To do so, we employ a Maxentropy principle, maximizing the uncertainty about q under the constraint that the expected value of p(q|f) be *f* (Equation (13)).

Before, when the expansion in delta distributions was used, each element of the base was associated with a single *f* value, equal to the property *F* evaluated on the corresponding level surface. A natural relaxation of this condition, while still fulfilling the requirement that the elements p(q|f) have the same level surfaces as F(q), is to demand that *f* be the *expected* value of F(q), when the average is weighted by p(q|f). If, after imposing this requirement, we insist that the base functions have no additional structure, then the shape of p(q|f) can be derived from the Maxentropy principle [13,14], in which the differential entropy H[p(q|f)] is maximized, conditioned to the restriction

(13) ∫dqF(q)p(q|f)=f.

The solution of this maximization problem is

(14) $$p\left(q|f\right)=\frac{e^{\beta F(q)}}{\int dq^{\prime }e^{\beta F(q^{\prime })}}=\frac{e^{\beta F(q)}}{Z_{0}\left(\beta \right)},$$

where the hyperparameter β is a function of *f*; that is, of the expected value of the property. The correspondence between each *f*-value and each β-value implies that both these parameters constitute valid tags to designate a member of the base. For this reason, in what follows, we use the two parametrizations interchangeably, depending on what we want to stress. We warn the reader that we pass from one to the other, tagging the elements of the base equivalently as p(q|f) or as p(q|β), understanding that there is a one-to-one mapping between *f* and β. In the nomenclature of Amari [15,16], the parameter appearing linearly in the exponent of the distribution (for us, β) is referred to as an *exponential* system of coordinates of the space of parameters. When *f* is used, the coordinates are called *mixed*. In equilibrium statistical mechanics, for example, β is often proportional to the negative of the inverse of the temperature, whereas *f* is proportional to the mean energy of a state—and of course, the two are related.

In the exponential family of Equation (14), all level surfaces of F(q) contribute to each p(q|f), but some are more relevant than others. If β is large and positive, p(q|f) is dominated by the level surface with maximal F(q) and rapidly dies out when we depart from it. The mean value of F(q) weighted by this p(q|β→+∞) is equal to the maximum of *F* on the simplex. If β=0, all level surfaces contribute uniformly. If β is large and negative, the level surface with minimal F(q) has maximal relevance, and the mean value of F(q) is equal to the minimum of *F* on the simplex. Therefore, β operates as a tuning knob that raises or lowers the relevance of different level surfaces, ranking them by the value of *F*. As happened when expanding the prior in a base of delta functions, the mean value of *F* shifts from its minimum to its maximum by adjusting the parameter. However, in contrast with the delta case, the expansion in exponential functions allows each element of the base to spread out to different level surfaces, allowing a whole diversity of surfaces to contribute to each 〈F|β〉.

### 2.5. The Estimation of the Property for Each Member of the Base

In this subsection, we show how the problem of estimating F(q) for each fixed β value is formally equivalent to obtaining the mean value of a certain physical quantity in statistical mechanics. In this analogy, the data *n* of the inference problem produce exponential distortions of the partition function of the Statistical Mechanical formulation.

The posterior distribution conditioned on a specific member β of the base is

(15) $$\begin{array}{ccc} p(q|n,\beta ) & = & \frac{p(n|q)\;p(q|\beta )}{p(n|\beta )}\\ & = & \frac{e^{\beta F(q)}\;\prod_{j}q_{j}^{n_{j}}}{\int dq^{\prime }\;e^{\beta F(q^{\prime })}\;\prod_{j}{q^{\prime }}_{j}^{n_{j}}}\\ & = & \frac{e^{\beta F(q)}\;\prod_{j}q_{j}^{n_{j}}}{Z(n,\beta )}. \end{array}$$

Replacing this expression in Equation (12)

$$\langle F|n,\beta \rangle =\frac{1}{Z(n,\beta )}\;\int dq\;F\left(q\right)\;e^{\beta F(q)}\;\prod_{j}q_{j}^{n_{j}},$$

where the posterior partition function Z(n,β) is calculated with the product of the prior distribution p(q|β) and the likelihood p(n|q). Just as in statistical mechanics [13], this integral is equal to

(16) $$\langle F|n,\beta \rangle =\partial_{\beta }\;\log Z\left(n,\beta \right).$$

Moreover, following the same reasoning used in statistical mechanics, the variance of the estimation reads

$$\langle \Delta F^{2}|n,\beta \rangle =\partial_{\beta }^{2}\log Z\left(n,\beta \right).$$

Importantly, in several applications, the partition function Z(n,β) can be calculated analytically. In those cases where an analytical treatment is impossible, the shape of Z(n,β) can often be well approximated by one-dimensional numerical integrals, which still enables the computation of 〈F|n,β〉.

### 2.6. Considering Different β Values

In this subsection, we demonstrate that out of all the possible β-values, the one that has maximal influence on the estimation process (or equivalently, the one that tags the most relevant base prior) depends on two independent factors. The first factor depends on the prior density g(β), comprising all the subjective elements of the inference process. The second factor is that for which the prior and posterior estimations of F(q) coincide and is entirely determined by the sampled data *n*. This result allows us to determine the role played by the different factors entering in the inference process.

Equation (10) states that the full estimation 〈F|n〉 results from integrating 〈F|n,β〉 in β, each contribution weighted by the evidence p(β|n). Thus, we now analyze this evidence.

When p(q|β) is given by Equation (14), the evidence of Equation (11) becomes

(17) $$\log p\left(\beta |n\right)\propto \log p\left(\beta \right)+\log p\left(n|\beta \right)=\log p\left(\beta \right)+\log\left[\frac{Z(n,\beta )}{Z_{0}\left(\beta \right)}\right],$$

where the constant of proportionality does not depend on β.

The first term of Equation (17) contains the prior expectations on β. The second one is the marginal likelihood p(n|β) and describes the way in which the data n favor a specific range of level surfaces. It is equal to the ratio of the posterior and the prior partition functions. The β value that makes the largest contribution to this ratio, henceforth called $\beta_{0}$, can be found by taking the derivative of the logarithm of the ratio and setting it to zero,

(18) $$\partial_{\beta }\log p\left(n|\beta \right){|}_{\beta_{0}}=\langle F|n,\beta_{0}\rangle -\langle F|\beta_{0}\rangle =0.$$

Therefore, $\beta_{0}$ is the parameter for which the prior and posterior estimations of the property coincide. This idea is illustrated in the last panel in Figure 1.

## 3. Applications to Different Properties

In this section, we apply the method to estimate the marginal probability of a bivariate distribution (Section 3.1), the amount of mutual information (Section 3.2), and the entropy (Section 3.3). All these quantities are examples of properties F(q) that have been extensively discussed in the literature [3,7,17,18,19]. Yet, as far as we know, all the methods developed so far—at least, those that perform decently in the severe undersampled regime—were idiosynchratically tailored to each specific property, whereas the method proposed here is general. In this subsection, we compare its performance to the more handcrafted approaches.

### 3.1. Marginal Probability

As a first example, we consider a system whose states are tagged by two labels: *x*, with many equiprobable states, and y∈{0,1}, with only two. The property we are interested in estimating is the marginal probability of y=1, denoted as $q_{1}$, defined as

$$q_{1}\left(q\right)=\sum_{x}q_{x}\;q_{1|x}=\frac{1}{k_{x}}\sum_{x}q_{1|x}\;,$$

were $k_{x}$ is the number of *x*-values. Since we assume all *x*-values have the same probability, here, q represents the collection of conditional probabilities ${\left\{q_{1|x}\right\}}_{x=1}^{k}$. This case constitutes a toy example, since the plug-in estimator ${\hat{q}}_{1}=n_{1}/n$ is unbiased and efficient, irrespective of the value of $n_{x}$. Still, we discuss this case to exemplify the procedure described in the previous section.

According to this procedure, the base functions used to expand the prior are solutions of a Maxentropy problem conditioned to have $q_{1}\left(q\right)=f$, which in exponential coordinates reads (Equation (14)),

(19) $$p\left(q|\beta \right)\propto \exp\left(\frac{\beta }{k_{x}}\sum_{x}q_{1|x}\right)\propto {\left(\prod_{x}e^{q_{1|x}}\right)}^{\beta /k_{x}}\;\propto \;\prod_{x}p\left(q_{1|x}|\beta \right).$$

For each β, this solution factorizes into independent terms. The exponential function favors either small or large values of $q_{1|x}$, depending on the sign of β. As the likelihood also factorizes $p\left(n|q\right)\propto \prod_{x}q_{1|x}^{n_{1x}}{\left(1-q_{1|x}\right)}^{n_{0x}}$, so does the posterior,

(20) $$p\left(q_{1|x}|n_{1x},n_{0x},\beta \right)=\frac{q_{1|x}^{n_{1x}}{\left(1-q_{1|x}\right)}^{n_{0x}}\;e^{\beta q_{1|x}/k_{x}}}{M(n_{1x}+1,n_{x}+2,\beta /k_{x})},$$

where M(a,b,z) is the confluent hypergeometric function [20]. Then, the partition function corresponds to $Z\left(n,\beta \right)=\prod_{x}M\left(n_{1x}+1,n_{x}+2,\beta /k_{x}\right)$. The prior partition function $Z_{0}\left(\beta \right)$ can always be obtained from Z(n,β) by setting n=0.

In Equation (20), the exponential factor is inherited from the prior $p(q_{1|x}|\beta )$. In general, in the method proposed here, each β biases the estimation towards the corresponding *f* value in order to get an unbiased estimate of the property when all the β values are considered.

With the partition function, we derive the estimate for the property $q_{1}$ of each member β of the family as

(21) $$\langle q_{1}|n,\beta \rangle =\frac{1}{k_{x}}\sum_{x}\langle q_{1|x}|n_{1x},n_{0x},\beta \rangle =\frac{1}{k_{x}}\sum_{x}\frac{\left(n_{1x}+1\right)}{\left(n_{x}+2\right)}\frac{M(n_{1x}+2,n_{x}+3,\beta /k_{x})}{M(n_{1x}+1,n_{x}+2,\beta /k_{x})}.$$

The parameter $\beta_{0}$ that makes a maximal contribution to the estimation is the one for which $\langle q_{1}|n,\beta_{0}\rangle =\langle q_{1}|\beta_{0}\rangle$, as established in Equation (18). This equations is often difficult to solve analytically. However, in the present case, it can be easily solved in the extreme undersampled regime ($n\ll k_{x}$) with no coincidences ($n_{x}\leq 1$), where there is a fraction $f_{1}=n_{1}/n$ of *x* states with y=1 and a fraction $\left(1-f_{1}\right)$ of states with y=0. This condition translates into

(22) $$\left(1-f_{1}\right)\frac{M(2,4,\gamma )}{3M(1,3,\gamma )}+f_{1}\frac{2M(3,4,\gamma )}{3M(2,3,\gamma )}=\frac{M(2,3,\gamma )}{2M(1,2,\gamma )}=\langle q_{1}|n,k_{x}\;\gamma \rangle ,$$

where $\gamma =\beta /k_{x}$. Taking into account the contiguous relations of the confluent hypergeometric function, this equation leads to $\langle q_{1}|n,\beta_{0}\rangle =f_{1}=n_{1}/n$, which is the naïve, plug-in, unbiased estimator. Usually for other properties, the plug-in estimator provides quite poor results in the severe undersampled regime, and there is no clear path to a low bias estimator. We believe that the proposed approach, focused on priors defined on the level surfaces, can be useful in these cases. In the two following subsections, we analyze other more challenging properties such as the mutual information and the entropy.

### 3.2. Mutual Information

The estimation of the amount of mutual information in a severely undersampled discrete system is a difficult task [2,21,22,23,24,25]. Several of the approaches developed previously [3,7] are based on separate estimators for the total and conditional entropies. Taking a different path, in a previous publication, we also developed a method that was focused on the dispersion of the conditional probabilities in relation to the marginal [8]. The performance was good, but still, the method was only applicable to mutual information.

In order to test the new method, we work with the same system introduced in the previous subsection. We assume $k_{x}\gg 1$, and all *x*-values have with equal probability $q_{x}=1/k_{x},\;\forall x$. The two states for the label y∈{0,1} are assumed to have equal marginal probabilities, qy=12,∀y (generalizations are explored in [8]). For this system, we want to find the mutual information I=I(X;Y), namely

(23) $$\begin{array}{cc} I & =\sum_{xy}q_{x}\;q_{y|x}\;\log\left(\frac{q_{y|x}}{q_{y}}\right)\\ \; & =\frac{1}{k_{x}}\sum_{x}\left[\log2+q_{1|x}\log q_{1|x}+\left(1-q_{1|x}\right)\log\left(1-q_{1|x}\right)\right]=\frac{1}{k_{x}}\sum_{x}I_{x}\left(q_{1|x}\right). \end{array}$$

As in the previous example, the property I(q) depends on the set of conditional probabilities $q={\left\{q_{1|x}\right\}}_{x=1}^{k_{x}}$.

The prior p(q|β) of Equation (14) reads

(24) $$p\left(q|\beta \right)\propto \exp\left[\beta I(q)\right]\propto \prod_{x}p\left(q_{1|x}|\beta \right).$$

Again, the prior factorizes. The individual factors are

(25) $$p\left(q_{1|x}|\beta \right)\propto \exp\left[\frac{\beta }{k_{x}}I_{x}\left(q_{1|x}\right)\right]\propto {\left[q_{1|x}^{q_{1|x}}\;{\left(1-q_{1|x}\right)}^{\left(1-q_{1|x}\right)}\right]}^{\beta /k_{x}}.$$

This prior resembles the beta distribution $\propto q_{1|x}^{\nu }\;{\left(1-q_{1|x}\right)}^{\nu }$ proposed in [8] but has non-constant exponents. Each posterior reads

(26) $$p\left(q_{1|x}|n_{1x},n_{0x},\beta \right)=\frac{q_{1|x}^{n_{1x}}{\left(1-q_{1|x}\right)}^{n_{0x}}\;e^{\beta I_{x}\left(q_{1|x}\right)/k_{x}}}{Z(n_{1x},n_{0x},\beta )}.$$

This functional form does not allow us to continue the problem in an analytically tractable fashion. Yet, the factorization makes the problem uni-dimensional, so it is easy to calculate the partition function for different pairs of $\left\{n_{1x},n_{0x}\right\}$ and the corresponding expectation values. Indeed, the partition function factorizes as $Z\left(n,\beta \right)=\prod_{x}Z\left(n_{1x},n_{0x},\beta \right)$.

**Figure 2:**
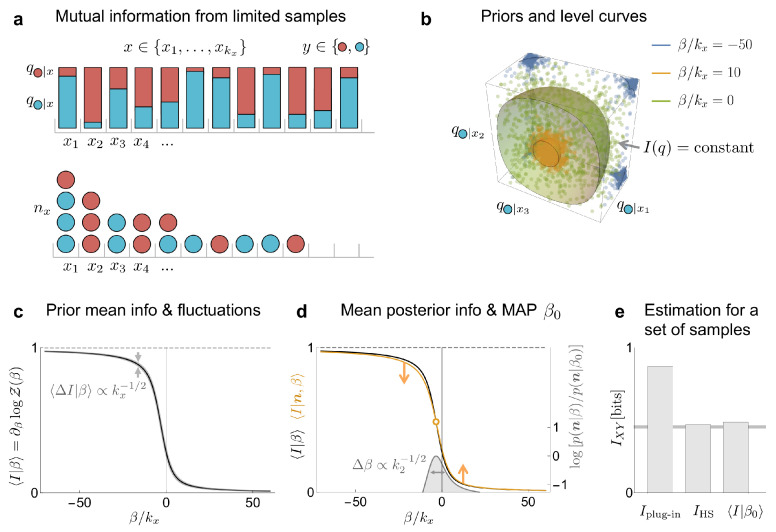
Toy example used to illustrate the estimation of the amount of mutual information in the same system as in Section 3.1. (**a**) The system is governed by a bivariate probability distribution, with states characterized by two labels: *x* and *y*. There are many *x* states, $k_{x}\gg 1$, and all are equally probable; that is, $q_{x}=1/k_{x}$. The variable *y* is binary, and its two values are depicted as red and blue, corresponding to 1 and 0, respectively. The conditional probabilities were sampled from a symmetric beta distribution with parameter 0.5. The goal is to estimate the mutual information $I_{XY}$ from *n* samples, with $n≳k_{x}$. (**b**) The level surfaces of the mutual information, as well as some sampled q-values, are displayed in a three-dimensional subspace of the full q-space, for different values of the hyperparameter β. (**c**) Prior mean mutual information as a function of the scaled hyperparameter $\beta /k_{x}$, and its fluctuations for $k_{x}=100$. (**d**) Prior and posterior mean mutual information as a function of the scaled hyperparameter $\beta /k_{x}$ for n=60 samples. The intersection of these curves corresponds to the MAP estimation $\langle I|\beta_{0}\rangle$. In gray, the posterior marginal evidence for the hyperparameter, whose width decreases as the square root of the number of states with at least two samples, $k_{2}$. (**e**) Comparison of the estimation of mutual information between different methods for the considered set of samples ($I_{\mathrm{HS}}$ is the estimator from [8]). The horizontal line corresponds to the true value of the mutual information.

The estimated value of the amount of mutual information of each β-value—the member’s guess— can be obtained with Equation (16), and the β-value that maximizes the marginal likelihood—the member’s evidence— from Equation (18). Interestingly, not all *x*-states contribute to the marginal likelihood,

(27) $$\log p\left(n|\beta \right)=\log\left[\frac{Z(n,\beta )}{Z_{0}\left(\beta \right)}\right]=\sum_{x}\log\left[\frac{Z(n_{1x},n_{0x},\beta )}{Z(0,0,\beta )}\right]$$

since the only non-vanishing terms are those in which the partition function associated to the *x* state is not proportional to the prior partition function; that is, $Z\left(n_{1x},n_{0x},\beta \right)/Z\left(0,0,\beta \right)$ varies with β. For mutual information, this condition is only met by *x* states with coincidences ($n_{x}\geq 2$). In fact, only when coincidences begin to appear ($n≳\sqrt{k_{x}}$) can the sampled data start to provide evidence about a non-vanishing mutual information. No such condition was found in the previous section, since there, every sampled *x*-state contributed to the estimation of the marginal probability. This discrepancy stems from the fact that, while the marginal probability depends on the first moment of the set $\left\{q_{1|x}\right\}$ of conditional probabilities, the mutual information relates to the second moment, or the amount of dispersion, of this set [8]. We show a toy example that illustrates this approach in Figure 2. In this example, the proposed method performs as well as some of the best known estimators (Figure 2e) designed for such undersampled regime.

### 3.3. Entropy

Finally, we consider the estimation of entropy, for which multiple estimators have been proposed in the past [1,3,4,5,6,26,27,28]. The entropy H(q) is defined as

(28) $$H\left(q\right)=-\sum_{x}q_{x}\log q_{x}.$$

Here, we use the natural logarithm for analytical convenience. We assume that the number of accessible states is unknown and that the number of effective states is quite large, so that exp[H(q)]≫1.

Once more, we expand the prior in a family whose members are tagged with β and use Equation (14) to express each member of the family as

(29) $$p\left(q|\beta \right)\propto \exp\left[\beta H(q)\right]=\prod_{x}q_{x}^{-\beta q_{x}},$$

where q belongs to the simplex $\sum_{x}q_{x}=1$. We can compare this prior to that used by NSB [3], a symmetric Dirichlet $p\left(q|\beta \right)\propto \prod_{x}q_{x}^{\beta -1}$. Although there are some similarities, the prior in Equation (29) has non-constant exponents, which hamper the analytical tractability. The posterior reads

(30) $$p\left(q|n,\beta \right)=\frac{\delta \left(\sum_{j}q_{j}-1\right)\;\exp\left[\beta H(q)\right]\;\prod_{x}q_{x}^{n_{x}}}{Z(n,\beta )}=\frac{\delta \left(\sum_{j}q_{j}-1\right)\prod_{x}q_{x}^{n_{x}-\beta q_{x}}}{\int dq^{\prime }\;\delta \left(\sum_{j}q_{j}^{\prime }-1\right)\prod_{x}{q^{\prime }}_{x}^{\left(n_{x}-\beta q_{x}^{\prime }\right)}}.$$

To calculate the member’s estimation 〈H|n,β〉 and the marginal likelihood p(n|β), the partition function Z(n,β) is needed.

**Figure 3:**
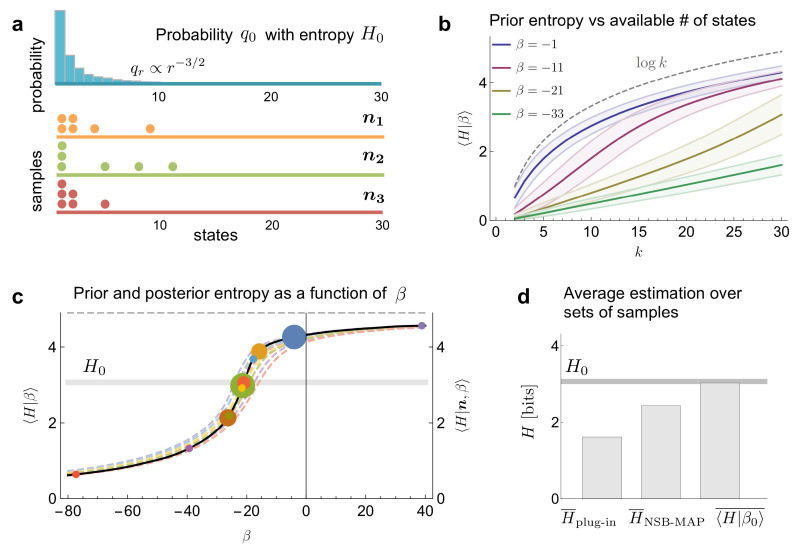
Entropy estimation in a toy example. (**a**) A distribution $q_{0}$ has ranked probabilities that decrease with a power law ($q_{r}\propto r^{-3/2}$) within a finite number of states (k=30). Distributions such as these represent a challenge for the estimation of entropy. Three possible sets of n=6 samples are displayed. (**b**) Prior mean entropy, plus/minus a standard deviation, as a function of the available number of states *k* for several values of the hyperparameter β. (**c**) Prior and posterior mean entropy as function of β for the different possible sets of multiplicities that can be obtained from n=6 samples (three examples shown in panel a, with matching colors). The intersections between prior and posterior mean entropy (MAP estimation) are marked with circles. The size of the circles is proportional to the likelihood of the corresponding multiplicity (a minimum size is imposed, for visibility). (**d**) Comparison of the average estimation of entropy between different methods over all the multiplicity sets (discarding the set with no coincidences, and the set with all samples in one state), weighting according to their likelihood. The horizontal line corresponds to the true value of the entropy.

We have found no evident way to solve the partition function analytically. Yet, its shape can be obtained by numerically integrating the partial convolutions that the delta over the simplex implies. With this result, we were able to obtain the prior and posterior estimates of the entropy, as well as the optimal β (Figure 3c). The MAP estimator of the entropy obtained with this procedure, even if numerical, was more accurate than the results derived from all previous methods we know of (Figure 3d).

## 4. Discussion

In this paper, we have proposed a novel Bayesian estimator to make an inference about a one-dimensional property of a high-dimensional system. Our work focused on the selection of the prior. The method takes advantage of the drastic reduction of dimensionality induced by the one-dimensional nature of the property of interest and employs a Maxentropy argument to eliminate the remaining indeterminate factors, thereby explicitly exploiting both what we know and what we do not know about the system. The approach is general in the sense that it can be applied to arbitrary properties and has excellent performance when compared to previous methods. Here, we discuss and summarize the main points of the paper.

### Conditions of Validity

When inferring the value of properties F(q), three sampling regimes are relevant. If the number of samples *n* is significantly smaller than $\sqrt{\exp[H(q)]}$, the sample is likely to contain no coincidences [1], and in this regime, no inference is possible. This study becomes useful when $\sqrt{\exp[H(q)]}≳n$, but still, the order of magnitude of *n* is not significantly larger than exp[H(q)]. For a still larger *n*, the estimation of the probabilities $q_{i}$ begins to be feasible, so it begins to make sense to consider the plug-in estimator.

### Identifying the Factors That Matter When Searching for the Prior

The Bayesian approach is argued to be the only rational way to infer the value of an unknown property. Yet, the success of Bayesian inference relies on an adequate choice of the prior. If an unjustified prior is used, only because there are no evident reasons to choose a specific prior, the result of the inference is not guaranteed to make sense. It has been argued that more than a drawback of the Bayesian approach, this sensitivity to the prior stresses the fact that it is impossible to make inference without assumptions [29]. Yet, it is often the case that selecting a prior is difficult, and this is problematic when the result is too sensitive to the choice, which is often the case in the severe undersampled regime. Without intending to diminish the relevance of priors, in this paper, we have shown that not all aspects of the prior distribution are relevant, and thereby we hope to focus the efforts of the selection process on the relevant aspects. For example, we have proved that all the modulations of the prior within the intersection between a level surface of the property and a level surface of the likelihood bear no consequences on the result of the inference. This observation, and the understanding that the prior distribution cannot depend explicitly on the sampled data, reduce the search of a high-dimensional prior p(q) to a one-dimensional prior g(f). Moreover, in many circumstances, even the detailed definition of g(f) may be more than required, although this statement only holds when the sampled data contain a non-negligible fraction of coincidences. When this condition holds, the data themselves select the range of *f*-values that actually contribute to the estimation. Therefore, only the variations of the prior distribution g(f) within this range actually matter.

### The Core of the Approach: Decomposing the Prior in a Conveniently Chosen Base of Priors

To make this selective role of the data explicit, and at the same time to be able to get analytical results, in this paper, we proposed the expansion of the prior distribution as a linear combination of base prior functions, each of which was required to have a different expected value of the property under study. By imposing no additional structure on the base priors (this requisite was instantiated by Maxentropy), the distributions of the base were derived to depend exponentially on the property under study. The most relevant element of the base (at least, inasmuchas the relevance was dictated by the data and not by g(f)) was shown to be the one for which the prior and posterior estimation of the property coincided (Equation (18)). This result is similar to the equality between the prior and posterior mean energy reported in the framework of statistical inference [30], and it relates to a scenario of parameter mismatch, as well as to the algorithm of expectation maximisation [31]. Even if the ratio of partition functions sometimes cannot be calculated analytically, the goal of this paper is to reveal the formal similarity between the two problems and thereby to prompt the reader to consider the battery of numerical algorithms that have been developed in expectation maximization to solve the type of one-dimensional problem posed here.

### Assessing the Effectiveness of the Method

In Section 3, we tested the performance of the method for three well-known properties, for which many estimators exist. Even though our method is general, it accomplished good results when compared to those produced by other methods specifically designed for each individual property. Interestingly, the performance was good even using a single β-value; namely, the one for which the marginal likelihood p(β|n) was maximal, without needing to integrate in β, and therefore, without needing to define the prior g(β). One fortunate ingredient was given by the fact that in those examples, as well as in many cases of interest, the property to be estimated was additive and symmetric in the components $q_{i}$. Properties with these characteristics can be written as $F\left(q\right)=\sum_{j}\varphi \left(q_{j}\right)$, for some function φ:R→R. When the effective number of states exp[H(q)] grows, the constraint imposed by the normalization condition becomes increasingly irrelevant, so the sampled $q_{i}$ can be assumed to be fairly independent of one another. In this case, the central limit theorem implies that the relative fluctuations of the property $\sigma_{F|n,\beta }/\langle F|n,\beta \rangle$ decay as the inverse of the square root of the effective number of states. Therefore, each member of the base makes a rather definite posterior guess 〈F|n,β〉 with minimal fluctuations, implying that even though the states p(q|β) spread all over the simplex, the posterior property is sharply defined within the state, as happened with the expansion in delta-like priors.

### Future Perspectives

There are several directions in which this work can be continued. From the strictly theoretical point of view, in Section 2.3, we argued that the optimal decomposition makes use of prior base distributions, the density of which is compressed inside a level surface, and there, the density is constant (Equation (5)). This choice, however, makes the obtainment of the partition function in analytical form unfeasible, since the integrals that need to be calculated are typically intractable. Therefore, we have opted to expand the prior in a base composed of distributions of fixed mean value. Future work should either demonstrate the optimality of this choice or suggest other possibilities.

It is also important to make a more systematic characterization of the relevant statistics in the data. For example, here, we have shown that the estimation of mutual information is only sensitive to states that have been sampled more than once. What is the relative effect of states sampled 2,3,⋯,s times? Which are the relevant statistics determining the estimation of other properties different from mutual information? More importantly, are there thresholds in the amount of sampled data for the possibility to make inference at all [30]? How do these thresholds depend on the property to be estimated?

From a more practical perspective, the excellent performance obtained for estimations of entropy and mutual information should also be evaluated in broader conditions; for example, for non-uniform marginals, or for sets with arbitrary cardinality. Moreover, the method should also be applied to a broader collection of properties, such as the estimation of Pearson correlation coefficients, slopes in linear regression, skewness, kurtosis, conditional mutual information [12], transfer entropy [32], etc.

### Conclusions

In summary, the method proposed here has the following attractive properties:

(a) It offers a general framework to select priors that is valid for arbitrary properties F(q);
(b) It performs well in the estimation of well-known quantities, as marginal distributions, mutual information and entropy, with performance comparable or even better than previous estimators individually tailored for each property;
(c) It reveals the formal similarity between inference problems and equilibrium statistical mechanics. In particular, the parameter β is analogous to the inverse temperature of statistical physics, and the two alternative expansions of the prior (in a delta-like or an exponential-like base) correspond to the microcanonical and the canonical ensembles, respectively.

## Data Availability

Not applicable.
